# Flash Pulmonary Edema: A Rare Presentation of Graves’ Disease

**DOI:** 10.7759/cureus.25833

**Published:** 2022-06-10

**Authors:** Maria Helena Rocha, André Conde, Luis Nogueira-Silva, Fernando Nogueira, Jorge S Almeida

**Affiliations:** 1 Internal Medicine, Centro Hospitalar e Universitário de São João, Porto, PRT; 2 Emergency Medicine, Centro Hospitalar e Universitário de São João, Porto, PRT; 3 Internal Medicine, Center for Research in Health Technologies and Information Systems (CINTESIS), Porto, PRT; 4 Internal Medicine, Centro Hospitalar Universitário de São João, Porto, PRT; 5 Medicine, Faculty of Medicine of University Porto (FMUP), Porto, PRT

**Keywords:** hyperthyroidism, thyroid storm, antithyroid antibodies, flash pulmonary edema, graves’ disease

## Abstract

Graves’ disease is the most common cause of hyperthyroidism. It has an autoimmune basis with the activating thyrotropin-receptor antibodies inducing thyroid hormone overproduction. The most common manifestations of hyperthyroidism are weight loss, fatigue, heat intolerance, tremor, and palpitations, but there are several other symptoms and signs associated with this condition. We report a case of a young woman who presented in the emergency room with acute onset of cough with mild hemoptysis and dyspnea at rest. She reported one month of insomnia, palpitations, and anxiety. The diagnostic investigation leads to the diagnosis of Graves’ disease in thyrotoxic crisis presenting with flash pulmonary edema. Therapy with propranolol and methimazole was instituted with remarkable clinical improvement.

## Introduction

Graves’ disease is the most common cause of hyperthyroidism and the incidence peaks between 30 and 50 years of age. This condition has an autoimmune basis with the activating thyrotropin-receptor antibodies inducing thyroid hormone overproduction. The diagnosis is based on the detection of low thyrotropin and high levels of free thyroxine and the presence of serum thyrotropin-receptor antibodies [1].

The most common manifestations of hyperthyroidism are weight loss, fatigue, heat intolerance, tremor, and palpitations, but there are several other symptoms and signs associated with this condition. Graves' disease has particular clinical manifestations, such as ophthalmopathy [1].

The cardiovascular effects of thyroid disease are some of the most profound and clinically relevant findings that accompany hyperthyroidism. The effects of thyroid hormones on the heart have been studied for several years and are now well known. Thyroid hormones decrease systemic vascular resistance and increase resting heart rate, left ventricular contractility, and blood volume. These hemodynamic changes can culminate in heart failure, known as high output heart failure. [2]

Flash pulmonary edema is a term used to describe a particularly severe presentation of acute heart failure with high output, usually associated with acute hemodynamic changes in cardiac or systemic conditions [3].

## Case presentation

A 46-year-old female patient with a previous history of recurrent spontaneous abortion was admitted to the emergency room for acute onset of cough with mild hemoptysis and dyspnea. She reported one month of insomnia, palpitations, and anxiety. No fever or other symptoms were present. She denied alcohol or drug consumption.

On physical examination, she had a blood pressure of 162/85 mmHg, heart rate of 100 per minute, respiratory rate of 30 per minute, a temperature of 36.8ºC, and peripheral oxygen saturation of 90%. The pulmonary and cardiac auscultation showed fine basal crackles and arhythmic tachycardia. She had neither peripheral edema nor signs of profound venous thrombosis. The arterial blood gas test revealed a type 1 respiratory failure (see Table 1).

**Table 1:** Arterial blood gas test in the emergency room with fiO2 of 45% fiO2: Fraction of inspired oxygen, pH: Potential of Hydrogen, pO2: Partial pressure of oxygen), pCO2: Partial pressure of carbon dioxide, HCO3: Bicarbonate, SO2: oxygen saturation

	Result	Normal range
pH	7.41	7.35-7.45
pO2	71.5 mmHg	> 60 mmHg
pO2/fiO2	158.8	> 300
pCO2	32.3 mmHg	35-45 mmHg
HCO3-	22.4 mEq/L	22-26 mEq/L
sO2	95%	95-100%
Lactate	1.33 mmol/L	< 1 mmol/L

**Figure 1:**
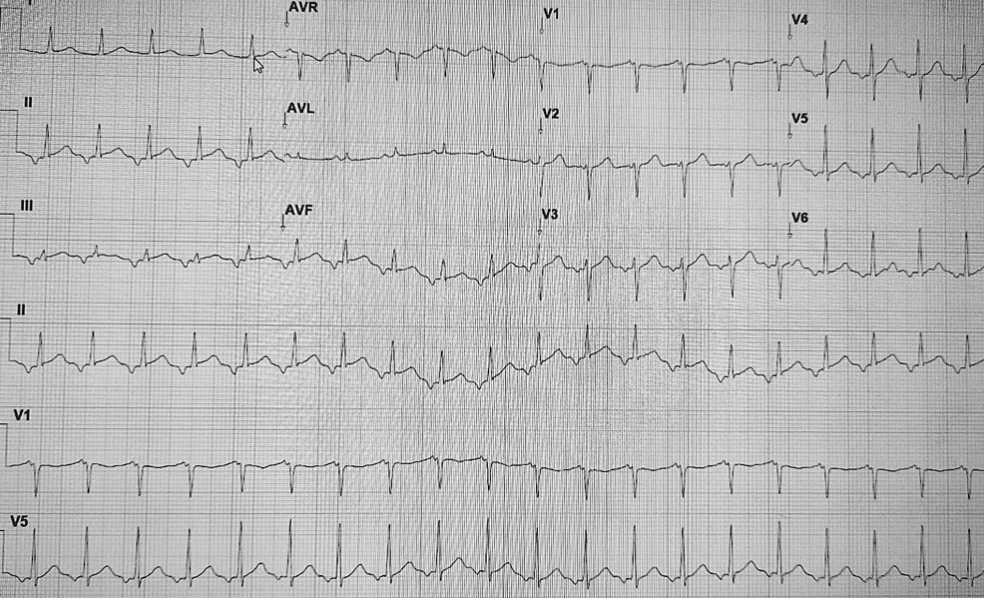
ECG taken in the emergency room

The electrocardiogram (ECG) showed an auricular tachycardia with a heart rate of 130 bpm and no other findings (see Figure 1). 

The treatment with oxygen and bronchodilators (salbutamol and ipratropium bromide) was started, while general blood tests and a thoracic contrasted pulmonary computer tomographic scan (CT angiography) was requested. This patient was admitted during the covid pandemic, which changed the available diagnostic features (for instance, a chest X-ray would take longer than a CT; the echocardiogram wasn't performed inside the covid-dedicated area). 

The general blood samples were normal for hemogram, renal function, hepatic enzymes, and c-reactive protein. The high-sensitivity cardiac troponin I (hscTn I) was slightly elevated at first (128,4 ng/L | N < 16 ug/L) but after one hour, had decreased and was nearly normal. Brain-type natriuretic peptides and d-dimers were normal (see Table 2). The reverse transcription-polymerase chain reaction testing for acute respiratory syndrome coronavirus 2 (SARS-CoV-2) infection was negative. 

**Table 2:** Laboratory findings

Test	Result	Normal range
Hemoglobin	13.1 g/dL	12-16 g/1dL
White blood cells	9.330 x 10^9^/uL	4-11 x 10^9^/uL
Platelets	293 x 10^9^/uL	150-400 x 10^9^/uL
Creatinine	0.41 mg/dL	0.51-0.95 mg/dL
Urea	33 mg/dL	10-50 mg/dL
Sodium	141 mEq/L	135-145 mEq/L
Potassium	4.5 mEq/L	3.5-5.1 mEq/L
Chloride	109 mEq/L	101-109 mEq/L
C – Reative Protein	10 mg/L	< 3 mg/L
Erythrocyte sedimentation rate	23 mm/hour	<25 mm/hour
Hs cardiac troponin	128.4 ng/L	<16 ng/L
Hs cardiac troponin (after 1h)	30 ng/L	<16 ng/L
Brain natriuretic peptide	70.6 pg/mL	<100 pg/mL
D-dimers	265 ng/mL	< 500 ng/mL

**Figure 2:**
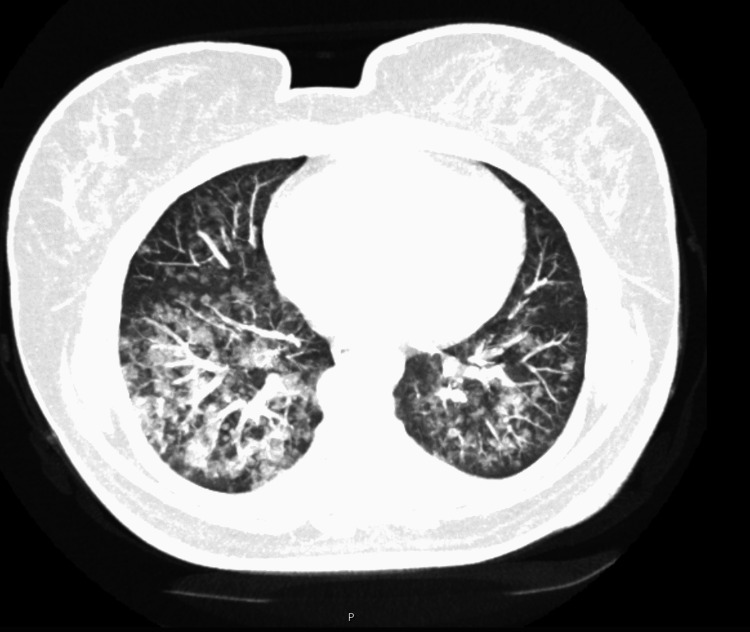
Contrasted pulmonary computer tomographic scan taken in the emergency room

The thoracic CT angiography showed bilateral ground-glass opacities with a perihilar distribution involving predominately the lower lobes; no signs of pulmonary thromboembolism nor evidence of alveolar hemorrhage were found (see Figure 2). At that time the patient remained stable with a peripheral oxygen saturation of 98% and a FiO2 of 35% via a Venturi mask and was admitted to an internal medicine ward for investigation.

In the first 24 hours, with the prescription of bronchodilators three times daily and furosemide intravenous 20 mg three times daily, the patient had clinical improvement with less cough and dyspnea. The auricular tachycardia (maximum 130 bpm) persisted with normal blood pressure, while no other abnormalities were found in the complete physical examination. The respiratory failure had resolved (see Table 3).

**Table 3:** Arterial blood gas test 24 hours after admission with fiO2 of 40% fiO2: Fraction of inspired oxygen, pH: Potential of Hydrogen, pO2: Partial pressure of oxygen), pCO2: Partial pressure of carbon dioxide, HCO3: Bicarbonate, SO2: oxygen saturation

	Result	Normal range
pH	7.41	7.35-7.45
pO2	239 mmHg	> 60 mmHg
pO2/fiO2	597	> 300
pCO2	35 mmHg	35-45 mmHg
HCO3-	22 mEq/L	22-26 mEq/L
sO2	100%	95-100%
Lactate	0.83 mmol/L	< 1 mmol/L

**Figure 3:**
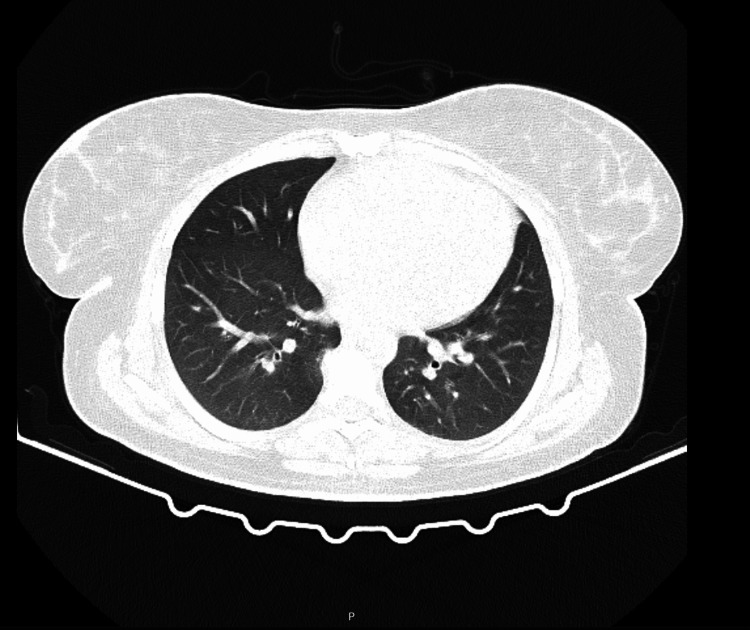
High-resolution computed tomography after 24 hours in the internal medicine ward

This clinical case was discussed in a multidisciplinary meeting on interstitial lung pathology, and it was decided to carry out a chest high-resolution computed tomography to better characterize the observed changes. The exam was repeated within the first 24 hours and showed remarkable improvement in the ground glass areas, and only mild densifications in some areas (see Figure 3).

After this surprising and rapid improvement without any specific treatment, the case was re-discussed. These transitory findings were only seen in flash pulmonary edema which is usually caused by an acute hemodynamic instability associated with cardiac or systemic conditions. 

The analytic study was extended, and showed a very low level of thyrotropin (TSH), increased free triiodothyronine (T3) and thyroxine (T4), and the presence of all three antithyroid antibodies (thyroid peroxidase; thyroglobulin; and TSH-receptor) (see Table 4). 

**Table 4:** Additional laboratory findings TSH: Thyroid-stimulating hormone

Test	Result	Normal range
Thyroid-stimulating hormone	0.001 uL/mL	0.35-4.94 uL/mL
T3	5.82 pg/mL	1.71-3.71 pg/mL
Free T4	1.99 ng/dL	0.70-1.48 ng/dL
Autoimmune findings		
Antithyroglobulin (Tg) and antithyroid peroxidase (TPO) antibodies: positive		
Anti-TSH (TRAb): 25.8 U/L (N: 0-1.8)		
Antinuclear antibodies (ANA) and Antineutrophil cytoplasmic antibodies (ANCA): negative		

**Figure 4:**
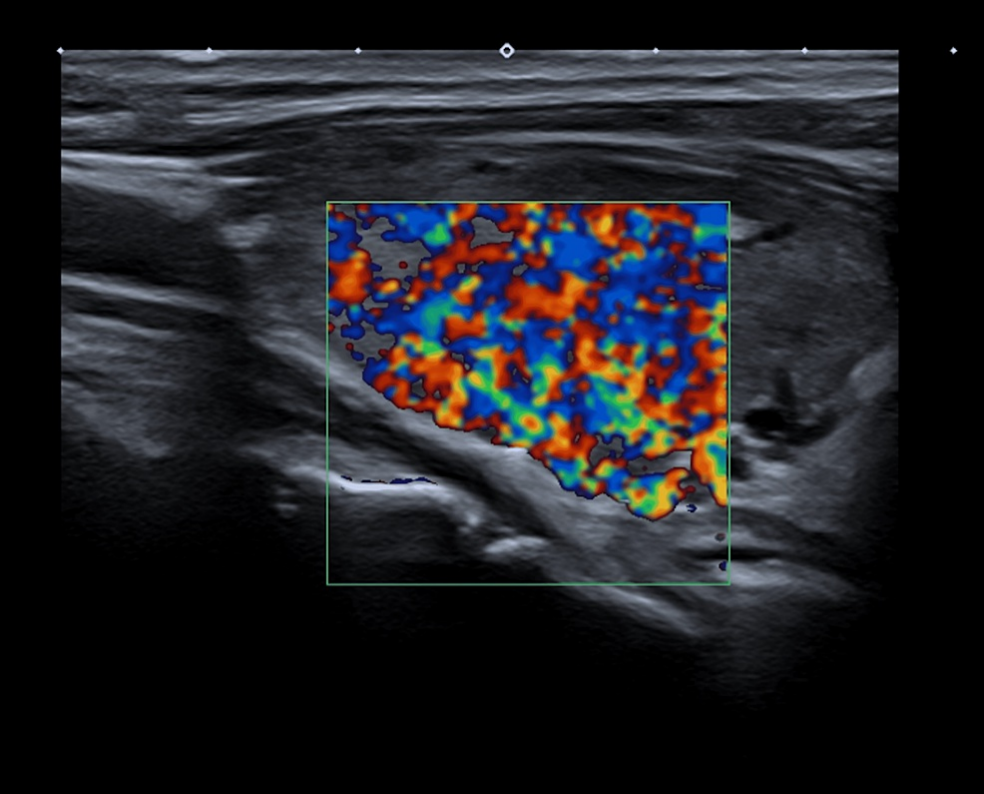
Thyroid ultrasonography with doppler

Thyroid ultrasonography was performed which showed enlargement of the thyroid gland with heterogenous and pseudonodular appearance, and the doppler analysis suggested increased vascularization compatible with thyroiditis (see Figure 4). 

The echocardiogram performed at that time showed no abnormalities. The diagnosis of Graves’ disease in thyrotoxic crisis was made. Therapy with propranolol and methimazole was instituted with heart rate normalization and a slight decrease of thyroid hormones after 72 hours with proper treatment. After one month in consultation, she reported no symptoms, non-occurrence of similar events, and had normal thyroid function. 

## Discussion

Graves’ disease is the most common cause of hyperthyroidism, presenting in more than 50% of patients with weight loss, heat intolerance, tremor, palpitations, tachycardia and ophthalmopathy [1]. Cardiovascular manifestations are one of the most studied and discussed over the years [2].

The hemodynamic changes induced by thyroid hormone can culminate in high-output heart failure with a cardiac output 50% to 300% higher than normal individuals [2]. Flash pulmonary edema is a term used to describe a form of acute decompensated heart failure associated to high-output, which can evolve to severe respiratory failure, intubation and death, mostly if the diagnosis is not considered and established quickly [3].

Three case reports [4-6] were found describing the association of flash pulmonary edema with Graves’ disease in thyrotoxic crises, two of them evolving to severe respiratory failure and intubation. 

Besides the normal echocardiogram that only was assessed in a more stable phase, the biochemical findings and the resolution of the radiologic abnormalities within 24 hours make an alternative diagnosis highly unlikely. Even with no other manifestations of Graves’ disease, the diagnosis pathway was fast and we believe that was the key to start the treatment early, preventing recurrences and unfavorable outcomes.

## Conclusions

Flash pulmonary edema as first manifestation of Graves' disease is extremely rare. As cardiopulmonary failure being the most common cause of death in thyrotoxic crisis, the diagnosis has to be considered when a healthy young patient presents with flash pulmonary edema without previous history of structural heart disease. The restoration of normal thyroid function usually reverses the abnormal cardiovascular hemodynamics leaving no cardiac sequelae. 
